# Clustering ball possession duration according to players’ role in football small-sided games

**DOI:** 10.1371/journal.pone.0273460

**Published:** 2022-08-25

**Authors:** Diogo Coutinho, Bruno Gonçalves, Timo Laakso, Bruno Travassos

**Affiliations:** 1 University of Maia, UMAIA, Maia, Portugal; 2 Department of Sports Sciences, Exercise and Health, University of Trás-os-Montes and Alto Douro, Vila Real, Portugal; 3 Research Center in Sports Sciences, Health Sciences and Human Development, CIDESD, CreativeLab Research Community, Vila Real, Portugal; 4 Departamento de Desporto e Saúde, Escola de Saúde e Desenvolvimento Humano, Universidade de Évora, Évora, Portugal; 5 Comprehensive Health Research Centre (CHRC), Universidade de Évora, Évora, Portugal; 6 Portugal Football School, Portuguese Football Federation, Oeiras, Portugal; 7 Department of Sport Sciences, University of Jyväskylä, Jyväskylä, Finland; 8 Department of Sports Sciences, University of Beira Interior, Covilhã, Portugal; Sport Sciences School of Rio Maior - Politechnic Institute of Santarem, PORTUGAL

## Abstract

This study aimed to explore which offensive variables best discriminate the ball possession duration according to players specific role (defenders, midfielders, attackers) during a Gk+3vs3+Gk football small-sided games. Fifteen under-15 players (age 13.2±1.0 years, playing experience 4.2±1.0 years) were grouped according to their positions (team of defenders, n = 5; team of midfielders, n = 7; team of attackers, n = 3). On each testing day (n = 3), each team performed one bout of 5-min against each team in a random order, accounting for a total of nine bouts in the following scenarios: i) defenders vs midfielders; ii) defenders vs attackers; iii) midfielders vs attackers. Based on video, a notational analysis process allowed to capture individual and collective actions. According to each playing position group, discriminant analysis was used to identify relevant variables that discriminate different ball possession sequences (short, medium, and long). The analysis revealed the existence of three clusters according to ball possession duration, classified as short sequence (~4 seconds), medium sequence (~10 seconds) and long sequence (~18 seconds). The number of touches per possession was the variable that discriminates the ball possession duration from all playing positions while passing actions were related to midfielders and attackers. In addition, different ball possessions sequences in the attackers were also discriminated by the number of players involved per possession. Accordingly, to increase the duration of the offensive phase during small-sided games, coaches should foster the players’ ability to stay on the ball, as it may amplify their opportunities to maintain the ball possession. In addition, coaches may also include reward rules to encourage midfielders and attackers’ passing actions and the number of attackers involved during the attack to promote longer ball possessions durations.

## Introduction

Ball possession has been received considerable attention under the last years [1]. From a practical point-of-view, the ball possession consists in all on and off-ball movements and actions developed in an attempt to create goal-scoring opportunities [2]. While different tactics and strategies may emerge when in possession (i.e., direct attack, counterattack or positional play), the success of teams such as Barcelona, Bayern Munich, Manchester City or the Spain National Team [3] had amplified the interest in the players’ and teams actions while attacking. For example, using the data for La Liga (2008–2009 season), it was found that possession and shots were a predictor of team success [4]. Also, when the ball possession was considered in relation to teams’ classification, it was identified that the teams ranked in the top presented more stable ball possessions than teams from the bottom [5]. Thus, it seems that the best teams are more able not only to maintain ball possession, but also held it for a longer period and for use it for the creation of shooting opportunities [5].

Considering this important view of ball possession, some studies have attempted to understand which factors may affect the ball possession duration. For example, it was found that the teams playing in the 1-4-2-3-1 formation presented in general higher ball possession duration than teams playing in the 1-4-2-2 [6]. When considering the contextual variables effects, it have been consistently identified that the ball possession increased when playing at home compared to away matches [7, 8], as well as when losing [5, 9, 10]. Using the ball possession to characterize the team’s performance according to opponents from different status level (i.e., playing against top or bottom teams) have also been addressed. For instance, higher ball speed and team effective playing space (i.e., space represented by the 10 outfield players) was found when playing against bottom teams compared to top teams [11]. In addition, playing against top teams contributed to a mean ball possession duration of ~28 seconds, while against weak teams this duration increased to ~37 seconds [11]. The time that a team holds the ball seems to reflect the complexity of their attacking process [12], and therefore, the ball possession duration had also become a focus of research.

When considering the ball possession duration during competitive matches, results have been inconsistent. While some studies reported that the chances of creating goal scoring opportunities emerges under lower ball duration possessions [3, 13, 14], other studies have shown the opposite [15, 16]. In this respect, the cultural behavioural seems to affect the team’s offensive strategy, and consequently, their tactical approach such as counterattack or the positional play. That is, the probability of scoring goals in less than 5 seconds possession in the English Premier League is of 0.7%, while the number and efficiency of attacks in the Spanish La Liga increases when possessions are longer than 12 seconds [15]. Thus, the ability to increase the ball possession duration, apart from the team tactics and strategy, seems to rely on the players’ technical ability. In fact, teams that has higher ball possession percentages are likely to perform more ~45% passes while also higher successful passes [17]. In this respect, some studies have been developed to consider which indicators describe different sequences of ball possession. For example, Merlin et al. [2] used four matches from the Brazilian Soccer Championship (2016) and identified three groups according to the ball possession duration and number of passes: i) short sequences (~11 seconds, ~2 passes per possession), ii) medium sequences (~27 seconds, ~5 passes per possession), and iii) long sequences (~56 seconds, ~12 passes per possession). Using the discriminant approach, the authors found that one of the variables that discriminate the duration of the possession was the number of passes to the last third [2]. Therefore, the ability to maintain the ball possession seems to be dependent upon the players’ ability to receive but mostly to pass the ball towards offensive zones.

When the players’ position is considered, a higher number of passes are performed by defenders, midfielders, and attackers during longer ball possession durations [17]. This is particular evident for defenders (passes completed: during lower ball duration, n = 30±12, during longer ball possession duration, n = 46±19) and midfielders (passes completed: during lower ball duration, n = 34±12, during longer ball possession duration, n = 55±20) as result of their role and higher participation in the build-up phase compared to the attackers (passes completed: during lower ball duration, n = 20±8, during longer ball possession duration, n = 25±11). In addition, increasing the ball possession duration seems to contribute towards more touches on the ball for the midfielders and forwards, while no differences between long and short ball possession durations for the defenders [8]. While it becomes clear the importance of the players’ technical ability to sustain longer ball possessions duration, no study to date has addressed which indicators may discriminate the time under possession according to the players’ playing position. Considering that different players’ roles require different perceptual and motor skills [18–20], it would be important to understand how the ball possession duration was affected according to these positions’ specificities Accordingly, coaches have been using small-sided games (SSG), which consists of game-based situations performed in smaller spaces, with adapted rules and often with a fewer number of players. Considering that different rules emphasize different information [21, 22], and consequently, different behaviours from the players, some research revealed that increasing the pitch area and playing in superiority stressed the passing actions [23], providing insightful information for coaches regarding on how to adjust the task constraints to emphasize ball possession. Despite the considerable amount of research exploring how different task constraints affects tactical behaviour of players and particularly ball possession, little is known how the manipulation of players capabilities and roles, constraints ball possession and the exploration of individual tactical actions. Therefore, this study aimed to explore which offensive variables best discriminate the ball possession duration (Short, Medium and Long) within players specific role (defenders, midfielders, attackers) during a Gk+3vs3+Gk football SSG.

## Methods

### Participants

Fifteen under-15 football players (age = 13.2 ± 1.03 years, height = 176.3 ± 7.5 cms, weight = 66.9 ± 8.7 Kg, playing experience = 4.2 ± 1.1 years) belonging to the same club participated in this study. The players were divided into three groups according to their playing position: a) defenders n = 5 (2 center backs and 3 fullbacks); b) midfielders n = 7 (3 center midfielders and 4 wide midfielders); c) attackers (3 forwards/strikers). Goalkeepers were part of the study but were excluded from the data analysis due to their positioning. According to the study aim, the data was inspected by analysing all ball possessions (n = 127) duration for each playing position (defenders, n = 49 ball possessions; midfielders, n = 38 ball possessions; attackers, n = 40 ball possessions). A written and informed consent was provided to the coaches, players, and by their legal guardians, as well as by the club, before the beginning of the study. All participants were notified that they could withdraw from the study at any time. The study protocol followed the guidelines and was approved by the local Ethics Committee of the Research Center in Sports Sciences, Health Sciences and Human Development (UIDB/4045/2020) and conformed to the recommendations of the Declaration of Helsinki.

### Task and procedure

Players were tested over three testing days, wherein each session they were exposed to the three conditions in random order: a) defenders team vs midfielders (DEF vs MID); b) defenders vs attackers (DEF vs ATT); and c) midfielders vs attackers (MID vs ATT). Therefore, players were exposed to a total of 9 SSG bouts (n = 3 per condition). Previous reports have shown that fullbacks present a distinct profile from central defenders, while also centre midfielders from wide midfielders [24, 25]. Despite that, when considering intra-sectorial training sessions, coaches often group the players’ according to their playing position (e.g., centre backs training with fullbacks to develop proper defensive alignment). Similarly, previous reports exploring positional relations and differences during competitive matches had also adopted only the three sectors [20, 26]. Based on the previous considerations, this study adopted a team configuration design that only accounted for the players’ playing position (e.g., grouping fullbacks with central defenders). The conditions were tested during a Gk+3vs3+Gk SSG performed in a 30x25m pitch [27, 28] and based on a 5-min duration each bout interspersed with a 10-min passive rest in between [28, 29]. A regulation ball size was used in all situations, and the SSG included two regular size goals (2.44 m x 7.32 m). The SSG were played with the official FIFA rules apart from: i) when the ball left the pitch (e.g., throw in) or a goal was score, the game restarted by the goalkeeper corresponding to the team in possession, ii) the offside rule was not applied. While the offside seems to affect the players’ positional behaviour during SSG [30], in this study, the main intention it was to understand how players individual and team actions were affected by the ball possession duration. In addition, previous reports suggested that SSG starting on the 4vs4 are more appropriate for analysing positioning performance [31], while this study explored individual and collective actions. In addition, these game-based formats were regular in the players’ normal training and have been adopted by other scientific research under similar aims [27–29, 32, 33]. No coach feedback or encouragement was allowed to avoid the potentially biasing effects of feedback on individual participant performance.

### Instruments

Players movements were recorded using a digital video camera (Sony HRZ-MC50E), positioned 7m above the ground, and forming an angle of approximately 45° with the longitudinal axis of the playing area. To assess the players and teams tactical behaviours, a notational system was created based on four categories [see 34, 35]: a) team behaviours; ii) players’ offensive individual actions, iii) players’ defensive individual actions, and iv), ball possession effectiveness (see Table 1 for independent variables and their description). These variables were captured by an expert analyst, and it were coded in line with previous research recommendations [see 34, 35]. To check the reliability of measurements, the same sample of SSG were re-analyzed after two weeks. Intra-observer reliability was calculated using the Cohen K index [36], and the values suggest a general adequate reliability of data (*K* = 0.931) as well as also good reliability values for each variable in analysis (see Table 1). At the end, an inter-observer reliability was calculated based on the analysis of 40 ball possessions, also suggesting adequate general inter-observer reliability of data (*K* = 0.866).

**Table 1:** Description of the independent variables.

Variables	Description	Reliability
**Team tactical behaviour**		
Players involved	The number of players that participate in that attack during ball possession	K = 0.942
**Participants’ offensive actions**		
Successful passes	Number of successful passes made by the team from one player to each other	K = 0.952
Diagonal and vertical passes	Number of diagonal and vertical passes a team completed in one attack	K = 0.933
Lateral and backward passes	Number of lateral and backward passes a team completed in one attack	K = 0.90
Rupture passes	A pass that split the last line of defence and plays a teammate through to shoot at the goal	K = 0.931
Unsuccessful passes	Number of wrong passes from one player to other player from the same team	K = 0.942
N. of Touches	Total number of all ball actions and events in which a player touch the ball	K = 0.884
Dribbles	Successfully completed dribbles made by a participant past layer an opponent	K = 0.892
**Players’ defensive actions**		
Ball recovery	A player successfully wins the ball back for his own team	K = 0.914
Ball Interception	A player successfully intercepts an opponent’s pass	K = 0.912
**Ball possession effectiveness**		
Goals	Number of successful shots ending in goal	K = 1
Shots	A team ends the ball possession with a missing shot, a shot resulting in a goal, or a shot saved by a goalkeeper.	K = 0.968

### Statistical analysis

A two-step cluster analysis with log-likelihood as the distance measure and Schwartz’s Bayesian criterion was undertaken to classify all ball possession sequences according to their duration variable. The original data were grouped into pre-clusters by constructing a cluster features tree, then the standard hierarchical clustering algorithm on the pre-clusters was used and provided a range of solutions with different numbers of clusters. The Schwartz’s Bayesian criterion (BIC), the BIC change value, the ratios of BIC changes and ratios of distance measures were calculated for each solution to find the optimal number of clusters. The quality of the clustering model was measured by the average silhouette coefficient, which is a measure of both cohesion and separation. It was assessed as follows: -1.0–0.2, poor model, 0.2–0.5, moderate-to-fair model; > 0.5, very good model [37]. Interpretation of the cluster results was described through calculating the mean values of the continuous variable of the duration of ball possession sequence. Afterwards, a descriptive discriminant analysis was used to identify which performance-related variables could best predict the obtained clusters. Variables were considered meaningful contributors to cluster group differences if their structure coefficients (SCs) in discriminant functions were higher than |0.30|. Validation of discriminant models was conducted using the leave-one-out method of cross-validation. The analyses were performed using IBM SPSS software (Armonk, NY: IBM Corp.).

## Results

**Fig 1:**
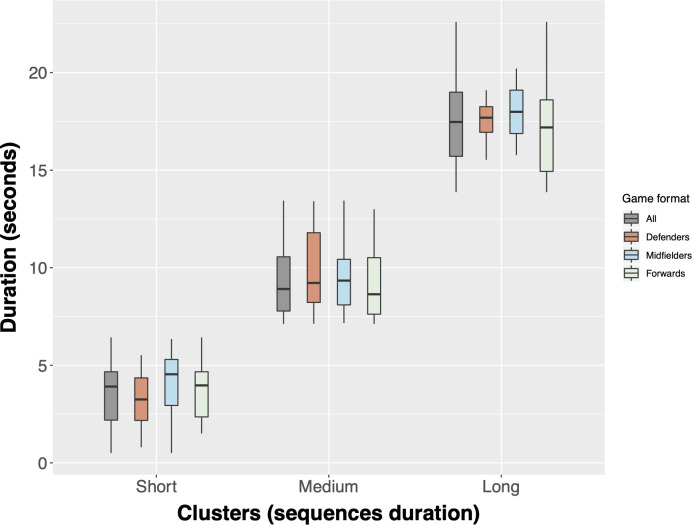
Box plot of the cluster analysis’ results according to the duration of the ball possessions sequences for each analysed game context. The lower and upper hinges correspond to the first and third quartiles (the 25th and 75th percentiles). The upper whisker extends from the hinge to the largest value no further than 1.5 * IQR from the hinge (where IQR is the inter-quartile range, or distance between the first and third quartiles). The lower whisker extends from the hinge to the smallest value at most 1.5 * IQR of the hinge.

An optimal number of three clusters were identified from a total of fifteen clustering solutions, with BIC = 97.22, change of BIC = -40.93, the ratio of BIC changes = 0.23 and ratio of distance measures = 2.58. The average silhouette coefficient was 0.7, indicating a very good cluster model. Fig 1 shows the labels and characteristics of three different clusters for all game scenarios. Considering all scenarios (n = 127 ball possessions), cluster 1 was composed of 66 possession sequences (52.0%) with 3.7±1.5 s duration and labelled by Short sequences; cluster 2 was composed of 49 possession sequences (38.6%) with 9.5±1.9 s duration and labelled by Medium sequences; and cluster 3 was composed of 12 possession sequences (9.4%) with 17.5±2.5 s duration and labelled by Long sequences.

**Fig 2:**
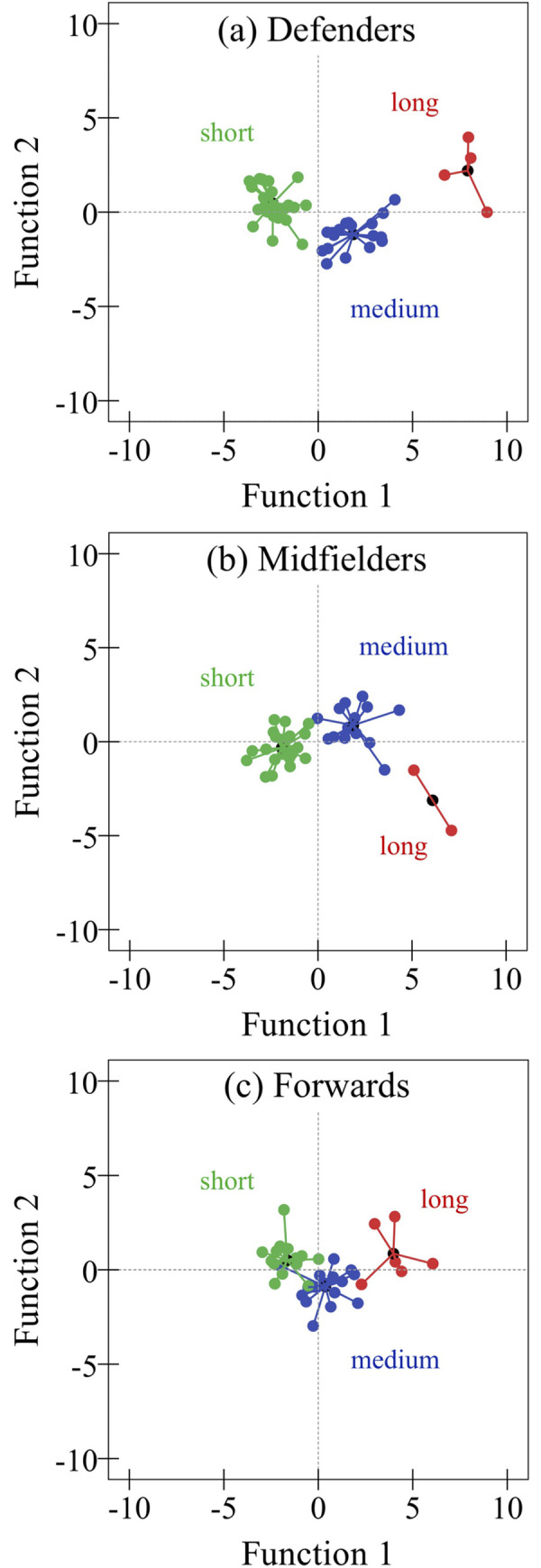
Territorial map of the discriminant functions for (a) Defenders, (b) Midfielders, and (c) Forwards’ game scenarios.

For the Defenders game scenarios, the short possession sequences (55.1% of the total possessions, n = 27) were characterized by 3.2±1.3 s, medium (36.7%, n = 18) by 9.8±2.0 s and long (8.2%, n = 4) by 17.5±1.5 s. The discriminant analysis generated two functions: function 1 explained 90.3% of variance (p < .001) and had the major contribution of the number of touches (SC range = 0.79) to the separation of ball possession sequences; function 2 explained (9.7%) (p < .01), where the number of players involved in possession (SC = -0.43), successful passes (SC = -0.35), and rupture passes (SC = 0.36) were the most important variables (see Table 2 for the descriptive and Fig 2A to territorial map of the discriminant functions).

**Table 2:** Means (±SD), discriminant function details and structure coefficients for the three clusters according to the defenders game scenarios.

Variables	Duration of the ball possessions (Defenders)			Structure coefficients	
	Short	Medium	Long	Function 1	Function 2
Players involved in possession	1.41±0.57	2.33±0.59	2.25±0.50	0.21	**-0.43**
Successful passes	0.30±0.47	1.50±1.04	1.75±0.50	0.25	**-0.35**
Lateral and backward passes	0.44±0.64	1.50±1.79	2.50±1.00	0.18	-0.07
Vertical and diagonal passes	1.11±1.31	2.17±1.42	1.75±1.26	0.09	-0.25
Rupture passes	0.00±0.00	0.11±0.32	1.00±1.15	0.21	**0.36**
Unsuccessful passes	0.30±0.47	0.17±0.38	0.25±0.50	-0.03	0.11
N. of touches	3.89±1.15	10.11±2.59	19.75±2.75	**0.79**	0.07
Dribbles	0.11±0.32	0.22±0.43	0.50±0.58	0.09	0.05
Ball recovery	0.04±0.19	0.17±0.38	0.50±1.00	0.11	0.07
Ball intercept	0.30±0.47	0.17±0.38	0.00±0.00	-0.07	0.01
Goals	0.41±0.50	0.33±0.49	0.75±0.50	0.04	0.18
Shots	0.70±0.47	1.00±0.59	1.50±0.58	0.14	0.03
% of Variance	n.a.	n.a.	n.a.	90.30	9.70
Eigenvalue	n.a.	n.a.	n.a.	10.19	1.10
Wilks’ Lambda	n.a.	n.a.	n.a.	0.04	0.48
Chi-square (χ^2^)	n.a.	n.a.	n.a.	126.20	29.59
Significance	n.a.	n.a.	n.a.	< .001	< .01

**Note:** Structure coefficients ≥|0.30| are bolded

In Midfielders, short possession sequences (55.3%, n = 21) had the duration of 4.1±1.7 s, medium (39.4%, n = 15) 9.5±1.8 s, and long (5.3%, n = 2) 18.0±3.1 s. Two functions were obtained from the discriminate analysis where the function 1, representing 85% of variance, was significant (p < .001). For function 1, Successful passes (SC = 0.32), Lateral and backward passes (SC = 0.30), Rupture passes (SC = 0.36), and N. of touches (SC = 0.66) were the most important variables to discriminate possessions, while Shots (-0.30) was the most important in function 2 (see Table 3 for the descriptive and Fig 2B to territorial map of the discriminant functions).

**Table 3:** Means (±SD), discriminant function details and structure coefficients for the three clusters according to the midfielders game scenarios.

Variables	Duration of the ball possessions (Midfielders)			Structure coefficients	
	Short	Medium	Long	Function 1	Function 2
Players involved in possession	1.62±0.67	2.20±0.68	2.00±0.00	0.15	0.24
Successful passes	0.57±0.68	1.87±1.41	3.50±2.12	**0.32**	-0.03
Lateral and backward passes	0.33±0.66	2.07±1.87	3.00±1.41	**0.30**	0.14
Vertical and diagonal passes	1.19±1.21	2.47±1.68	5.00±4.24	0.26	-0.13
Rupture passes	0.00±0.00	0.87±1.30	3.00±0.00	**0.36**	-0.27
Unsuccessful passes	0.33±0.48	0.53±0.52	0.50±0.71	0.07	0.10
N. of touches	4.48±1.94	10.80±2.60	15.00±2.83	**0.66**	0.23
Dribbles	0.14±0.36	0.20±0.41	0.00±0.00	0.00	0.13
Ball recovery	0.19±0.40	0.07±0.26	0.00±0.00	-0.08	-0.04
Ball intercept	0.14±0.36	0.07±0.26	0.00±0.00	-0.06	-0.01
Goals	0.24±0.44	0.00±0.00	0.00±0.00	-0.14	-0.15
Shots	0.57±0.51	0.33±0.62	1.00±1.41	-0.01	**-0.30**
% of Variance	n.a.	n.a.	n.a.	86.00	14.00
Eigenvalue	n.a.	n.a.	n.a.	5.85	0.95
Wilks’ Lambda	n.a.	n.a.	n.a.	0.07	0.51
Chi-square (χ^2^)	n.a.	n.a.	n.a.	75.18	19.39
Significance	n.a.	n.a.	n.a.	< .001	.08

**Note:** Structure coefficients ≥|0.30| are bolded.

Finally, in the forwards game scenarios, short (n = 18), medium (n = 16), and long possession (n = 6) sequences lasted, respectively, the duration of 3.9±1.6 s (45.0%), 9.3±2.1 (40.0%) and 17.4±3.2 (15.0%). The discriminant analysis generated two functions: function 1 was significant (p < .001), explained 87.1% of variance, and where the variables players involved in possession (SC = 0.39), successful passes (SC = 0.35), lateral and backward passes (SC = 0.32), vertical and diagonal passes (SC = 0.31) and number of touches (SC = 0.80) were the most important variables (see Table 4 for the descriptive and Fig 2C to territorial map of the discriminant functions).

**Table 4:** Means (±SD), discriminant function details and structure coefficients for the three clusters according to the forwards game scenarios.

Variables	Duration of the ball possessions (Forwards)			Structure coefficients	
	Short	Medium	Long	Function 1	Function 2
Players involved in possession	1.22±0.55	2.13±0.72	2.50±0.55	**0.39**	**-0.47**
Successful passes	0.44±0.70	1.06±1.12	2.33±1.03	**0.35**	0.04
Lateral and backward passes	0.28±0.96	1.06±1.73	3.17±2.40	**0.32**	0.11
Vertical and diagonal passes	1.00±1.33	1.81±1.47	3.83±2.64	**0.31**	0.08
Rupture passes	0.06±0.24	0.63±1.09	1.33±1.51	0.26	-0.08
Unsuccessful passes	0.11±0.32	0.38±0.62	0.67±0.82	0.19	-0.08
N. of touches	4.39±1.79	9.44±3.42	16.83±3.43	**0.80**	-0.13
Dribbles	0.06±0.24	0.69±0.79	1.00±1.26	0.25	-0.27
Ball recovery	0.06±0.24	0.13±0.34	0.17±0.41	0.07	-0.06
Ball intercept	0.11±0.32	0.13±0.34	0.17±0.41	0.03	0.01
Goals	0.28±0.46	0.31±0.48	0.33±0.52	0.02	-0.02
Shots	0.56±0.51	0.69±0.70	0.83±1.17	0.07	-0.03
% of Variance	n.a.	n.a.	n.a.	87.05	12.95
Eigenvalue	n.a.	n.a.	n.a.	3.95	0.59
Wilks’ Lambda	n.a.	n.a.	n.a.	0.13	0.63
Chi-square (χ^2^)	n.a.	n.a.	n.a.	63.92	14.33
Significance	n.a.	n.a.	n.a.	< .001	.28

**Note:** Structure coefficients ≥|0.30| are bolded.

## Discussion

This study aimed to explore which offensive variables best discriminate the ball possession duration (Short, Medium and Long) within players specific role (defenders, midfielders, attackers) during football small-sided games.

Over the last years, there has been an increased body of research attempting to explore and relate ball possession to teams’ success during competitive performances [2, 5, 10, 15], mainly as the result of the positive results achieved by professional teams playing under possession play (e.g., Barcelona, Manchester City…) [3, 38]. While some studies showed that counterattacks and directed attacks, which consequently are characterized by lower duration, may amplify the opportunities to score [3], recently, it seems that the higher ball durations enhanced scoring chances [6]. In this respect, some studies have attempted to distinguish short from long ball possession durations. For instance, Jones, James [39] considered three categories of ball possession duration (i.e., 3 to 10 seconds, 10 to 20 seconds, and > 20 seconds), and found that more successful teams spend more time on possession (i.e., > 20 seconds) compared to unsuccessful teams (~10% more) [39]. Recently, other study adopted the discriminant analysis technique to classify the ball possession duration from 4 official matches of the Brazilian National Championship, and the clusters return three clusters according to ball duration and number of passes: short (~11 seconds, ~2 passes), medium (~27 seconds, ~ 5 passes) and long duration (~56 seconds, ~12 passes) [2]. The present study adopted a similar approach as the one adopted by Merlin et al. [2], showing as well three clusters, however with different configurations. Accordingly, most ball possessions were of short duration (~52%, ~4 seconds duration), followed by the medium sequences (~39%, ~10 seconds duration) and lastly the long sequences (~9%, ~18 seconds duration). While the study from Merlin, et al. [2] explored Gk+10vs10+Gk situations, this study focused on Gk+3vs3+Gk. These differences may explain the difference between both studies, as lower game-based formats are likely to emphasize more the technical actions rather the tactical ones [31], and consequently, having lower duration. In fact, a previous study comparing Gk+3vs3+Gk with a larger format composed by Gk+6vs6+Gk showed higher ball possession durations in the last one [40].

In this study, the number of touches per player were the variable that best discriminate the short, medium and long ball possessions duration independently of the playing position. In this respect, players performed in average 4 touches for short ball possessions, 10 for medium ball possessions and 20 for long ball possession sequences. Using the same number of players, other study found an average of ~7/8 touches per ball possession [40]. In this point-of-view, it seems important to distinguish different ball possession durations as it would allow to improve knowledge regarding to which indicators may better discriminate longer ball possessions durations. Based on our results, longer ball possessions seem to be dependent upon the players ability to stay on the ball. In fact, it has been highlighted that players’ technical skills are crucial to increase ball possession [1], and so, it is not surprising that higher ball possessions durations entails a higher number of touches on the ball.

Apart from number of touches, players involved per possession, successful passes and rupture passes were identified as variables that discriminate ball possession durations in function 2 for the defenders. Defenders have the two main roles: a) start the build-up process by being able to progress on the field with the ball or performing penetrative (i.e., rupture passes), while b) when defending protecting the goal and avoid goal-scoring opportunities [41]. The results found in this study are like those exploring the difference between low and long ball possession durations in elite players from English Premier League [8], which also found differences in the number of passes and successful passes in this playing position. Modern football, where offensive strategy characterized positional play has become more used [42], requires central defenders that possess well developed passing skills to be able to find teammates between opposing team defensive lines [43]. In fact, research has suggested that defenders have increasing the passing accuracy over the last years [44, 45]. In addition to successful passes, this study adds the longer ball possessions are also characterized by the defender’s ability to performing rupture passes during Gk+3vs3+Gk SSG, possibly, because it would allow them to find more space to progress and stay on the ball.

Studies showed that longer possessions led to a higher number of passes, touches per possession, shots, dribbles and final third entries [8]. When we consider the midfielders playing role, this playing position is characterized by players that can control the game pace and responsible for progressing with the ball to further zones in the pitch throw travelling with, performing penetrative passes or receiving the ball between defensive spaces [18, 20, 28]. Therefore, it is not surprising that the variables that discriminated the longer from shorter sequence were mainly related to passing actions (i.e., successful passes, lateral, backward, and rupture passes) and the number of touches per possession. Previous reports comparing positional roles technical actions under short and longer ball possession durations had also shown that successful passes, passes received and final third entries were the variables that discriminate the ability to stay on the ball [8]. In addition to this, study exploring network metrics had shown the midfielders as the players with higher values of centrality, which represents nuclear players to the team in terms of the ball flow (i.e., receiving and passing) during both SSG [46] and competitive matches [18]. In this respect, coaches may promote different SSG variations in the midfielders roles to couple their passing actions according to dynamic information from the environment, such as varying pitch configurations [47] or even manipulate perceptual demands to affect the passing direction [48].

The discriminant results from the attackers also revealed that the variables that better discriminate the ball possession duration were mostly related to passing actions (successful passes, lateral, backward, vertical and diagonal passes), while also the number of players involved and the number of touches. Usually, the attackers are characterized by refined technical skills to cope with the defenders’ pressure and face outnumbered situations [49, 50]. Considering that the prevalent intention from this playing position is to score goals, they usually seem to focus more on the surrounding information (e.g., space between defenders, distance to the target, goal location) [51] and engage in more individual situations such 1vs1 to create space [20]. However, at modern football where there seems to be an emergent trend to defend more compact and with a high number of defenders [52], which may model the attacker’s role towards deceiving the opponents’ by moving to other areas or holding the ball until receiving pressure to then be able to find a free man. As consequence it may contribute towards longer ball possessions, while shorter ball possessions may be characterized by more individual actions. In fact, the study of Bradley et al. [8] that compares players’ role performance during competitive matches as result of ball possession duration found increases in the number of passes, touches on the ball in addition to also being more tackled. This may support the suggestion the important role of strikers in holding the ball during longer ball possession durations. As expected, the previous study found higher mean values of passing actions in longer ball possessions (i.e., ~25 passes) than in the present study (i.e., ~3 passes). Thus, it seems important that during the practices, coaches design small game scenarios as it seems to amplify technical actions embbebbed under certain game rules [27], while also, providing more large game scenarios that simulate the complexity in terms of number of players, decision-making and available opportunities for action as those found during competitive performances [53].

Overall, this study showed which variables best discriminate players’ performance during a Gk+3vs3+Gk SSG when considering different ball possession durations. In general, the number of ball touches was considered as a variable that discriminate the ball possession duration in all playing positions. In this respect, coaches may foster the development of players’ ability to stay on the ball by developing perceptual (e.g., analysing the environment, perceiving teammate and opponents’ movements) [51] while also motor skills as the frequency of touching the ball as it may provide competitive advantage during 1vs1 situations [54]. In this respect, adopting smaller game formats seems to be beneficial [32], however, again it is important to be aware that larger formats may also help players to be fine-tuned to the same perceptual-motor landscape that they face during competition. Additionally, designing teams based on players’ from the same profile may help them to develop other skills usually less performed, such as the shots on defenders [28]. As so, when considering the increasing importance attributed to ball possession and its relationship with team success [2, 5, 10, 15], coaches may be aware that to foster the ability to maintain possession may design tasks to emphasize the number of touches for all playing positions, while also a variety of passing actions (i.e., lateral, backward, vertical and diagonal passes for attackers and midfielders, while also rupture for the midfielders). For that purpose, coaches may adopt rewarding tasks that encourages the players to pass the ball (for midfielders and attackers) and to involve more players (for the attackers). As an example, performing at least one rupture pass would contribute to a doble goal, while the number of goals scored by the attackers would be correspondent to the total number of players’ that participated in the offensive process (e.g., 2 attackers = 2 goals, 3 attackers = 3 goals).

Whilst this study provides important and practical implications for team composition criteria according to players’ position and ball possession duration, some limitations should be acknowledged. Firstly, this study adopted an approach where the players were grouped according to the team sector (i.e., defenders, midfielders and attackers), and based on a real club case scenario, which may refrain from achieving stronger inferences. In addition to this, the teams have only faced opposing players team compositions (e.g., midfielders vs defenders and midfielders vs attackers), while adding comparison between two teams from the same playing position (i.e., midfielders vs midfielders) may bring new variables into the discussion. In this respect, to develop a more robust data analysis, further studies should consider increasing the sample size, considering a balanced number of players between players’ positions, while also accounting with participants from different environmental contexts and age groups as it seems to contribute to different ball possession strategies. It would also be important to compare different categories within the playing positions (e.g., fullback vs central defenders; defensive midfielders vs wide midfielders; forwards vs wingers) as each category have unique characteristics that would refine the understanding on which performance indicators would best discriminate different ball possessions durations according to the specific playing positions.

## Conclusion

Overall, this study allowed to identify which variables best discriminate different ball possession durations (i.e., short, medium, and long) according to the players’ positional role (i.e., defenders, midfielders and attackers). The number of touches was one of the variables that mostly distinguish between different ball possession sequences for all playing positions. Interestingly, this was the only discriminating variable for the defenders, which may be related to their role of starting the offensive phase. Apart from the number of touches, the midfielders were also described by the type of passing actions performed (i.e., successful passes, lateral, backward and rupture passes), that may result from their main role in the team behaviour linking defenders to the attackers. Therefore, longer ball possessions depend on their ability to successfully maintain the ball by using the pass as the preferred option. The attackers were described by the number of touches, passing actions and the number of players involved in the possession. This study adds important information regarding the variables that discriminate different ball possession sequences according to the players’ role and may help coaches develop appropriate behaviours that would allow the players to increase the time with the ball.
